# Supplementary material for: Comparison of patient perceptions of primary care quality across healthcare facilities in Korea: A cross-sectional study
Source: PLoS One. 2020 Mar 10;15(3):e0230034. doi: 10.1371/journal.pone.0230034 (PMC7064208; doi:10.1371/journal.pone.0230034)
Supplement: S1 File — (DOCX) [file pone.0230034.s002.docx]

**S1 File. Questionnaire (English)**

**■ General Information**

**1. Gender** □ Male □ Female

**2. Age** _________________

**3. Status** □ Faculty □ Staff □ Student

**4. Which of the below best describes the current economic status of you and your family?**

□ High □ Upper-middle □ Middle □ Lower-middle □ Low

**5. In general, how would you rate your overall health?**

□ Very good □ Good □ Fair □ Poor □ Very poor

**6. Choose all your current health problems.**

□ None

□ Frequent colds

□ Frequent digestive symptoms (indigestion, heartburn, stomachache, etc.)

□ Headache, dizziness

□ Insomnia, sleep problems

□ Allergic rhinitis, atopy, asthma

□ Hypertension

□ Hyperlipidemia

□ Diabetes

□ Hyperuricemia, gout

□ Heart disease

□ Arthritis, joint pain

□ Depression

□ Anxiety

□ Chronic hepatitis (hepatitis B, C)

□ Cancer (present or past)

□ Other _____________________

**7. Do you or your family regularly visit the doctor for chronic medical conditions (e.g., hypertension, diabetes)? If true, who is in your family?**

□ None

□ Myself

□ One or more members of my family except for me

□ Myself and my family

**8. Are you or any of your family members a medical doctor (excluding dentists and oriental doctors)?**

□ No □ Yes

**9. Do you have a regular doctor who continuously takes care of your health and provides comprehensive counseling?** □ No □ Yes

**10. How often did you see the doctor (excluding a dentist or oriental doctor) for your health problems last year?**

□ Never

□ 1–3 times

□ 4–6 times

□ 7–12 times

□ ≥13 times

**11. How much did you spend on your medical care at the clinic/hospital last year? (except for medication and health checkup costs)**

□ < 250,000 KRW

□ 250,000–500,000 KRW

□ 500,000–750,000 KRW

□ 750,000–1,000,000 KRW

□ >1,000,000 KRW

**■ Primary Care Assessment**

**12. Please rate how much you agree with the following statements on a 5-point scale, based on your experience at each facility below. (You can skip those that you have never visited.)**

♦ 5-point scale: 1. Strongly agree 2. Agree 3. Neutral 4. Disagree 5. Strongly disagree

|  | **SNUHSC** | **Community**  **clinic** | **Large hospital** |
| --- | --- | --- | --- |
| When I have a new health problem, I will visit the facility first. |  |  |  |
| The facility is easy to access geographically and temporally. |  |  |  |
| The out-of-pocket cost is appropriate and affordable. |  |  |  |
| The doctor provides comprehensive care for various health problems. |  |  |  |
| I will visit the facility for basic health care such as periodic physical exam and blood tests (e.g., blood sugar, cholesterols). |  |  |  |
| I will visit the facility for simple medical procedures (e.g., wound disinfection, closure, and dressing). |  |  |  |
| I will consult the doctor for health counseling and education on healthy lifestyle (nutrition, exercise, smoking, alcohol use, etc). |  |  |  |
| I will visit the facility for a regular general health checkup before going somewhere else. |  |  |  |
| The doctor knows my complete medical history and health states. |  |  |  |
| The doctor suggests me go to a specialist or special service when additional care is required. |  |  |  |
| The doctor tries to listen to and understands my words and questions well. |  |  |  |
| The doctor provides an easy and detailed explanation of my health states and test results. |  |  |  |
| The doctor is interested in my mental health problems as well as physical health problems. |  |  |  |
| The doctor knows and has a concern about my family and living environment. |  |  |  |
| The doctor is active in promoting the community health (health courses, home visits, etc.) |  |  |  |
| The facility surveys and reflects patients' opinions to provide better health care. |  |  |  |
| I can trust the doctor's decisions on treatment. |  |  |  |
| Overall, the health care service provided is satisfactory. |  |  |  |
