# Supplementary material for: Comparison of patient perceptions of primary care quality across healthcare facilities in Korea: A cross-sectional study
Source: PLoS One. 2020 Mar 10;15(3):e0230034. doi: 10.1371/journal.pone.0230034 (PMC7064208; doi:10.1371/journal.pone.0230034)
Supplement: S2 File — (DOCX) [file pone.0230034.s003.docx]

**S2 File. Questionnaire (Korean)**

**■ 일반 사항**

**1. 성별** □남 □여

**2. 나이**만­­­­­________세

**3. 직업**□교원    □직원  □학생

**4. 본인 및 가족의 경제수준은 대체적으로 어떠합니까?**   □상 □중상 □중 □중하 □하

**5. 본인의 전반적인 건강상태는 어떠합니까?**

 □아주 좋음 □좋음 □보통 □나쁨 □매우 나쁨

**6. 평소 겪는 건강문제를 모두 선택하세요.**

 □없음   □잦은 감기   □잦은 소화기 증상(소화불량/속쓰림/복통 등)

 □두통, 어지럼증   □불면증, 수면문제 □알레르기성 비염/아토피/천식

 □고혈압   □고지혈증   □당뇨

 □고요산증/통풍   □심장질환   □관절염/관절통

 □우울증   □불안증   □만성간염(B형, C형)  □암(현재 또는 과거) □기타

**7. 본인 또는 가족분이 고혈압/당뇨 등 만성질환으로 의료기관을 정기적으로 이용하십니까? 누구입니까?**     □없음 □본인 □가족 □본인 및 가족

**8. 본인 또는 가족 중에 의사가 있습니까? (치과의사, 한의사는 제외)** □없음  □있음

**9. 평소 귀하의 건강을 지속적으로 관리해 주고 건강문제를 종합적으로 상담할 수 있는 의사가 있습니까?**

 □예   □아니오

**10. 지난 1년간 본인의 건강문제로 의료기관을 방문한 횟수는 총 몇 번입니까?**(치과, 한방 병의원 제외)

 □없음

 □1–3회

 □4–6회

 □7–12회

 □13회 이상

**11. 지난 1년간 본인의 진료/검사/치료 비용으로 "병원"에 지출한 비용은 대략 어느 정도입니까?** (약제비, 건강검진비용은 제외)

 □25만원 미만

 □25–50만원

 □50–75만원

 □75–100만원

 □100만원 이상

**■ 일차의료 서비스 평가**

**12. 평소 다음 의료기관을 이용한 소감을 바탕으로 평가한 점수를 1–5 숫자로 해당 칸에 작성하십시오.**

 (이용한 적이 없는 의료기관은 해당 칸을 건너뛰실 수 있습니다.)

**◈ 점수 설명:  1.전혀 그렇지 않다  2.그렇지 않다   3.보통   4.그렇다   5.매우 그렇다**

|  | **서울대**  **보건진료소** | **동네의원** | **대형병원** |
| --- | --- | --- | --- |
| 새로운 건강문제가 생겼을 때 가장 먼저 방문하겠다 |  |  |  |
| 가까이 위치하고, 원하는 시간에 쉽게 진료 받을 수 있다 |  |  |  |
| 진료비, 검사비가 적절하고 부담 없다 |  |  |  |
| 다양한 건강문제를 종합적으로 관리 받을 수 있다 |  |  |  |
| 진찰, 혈액검사 등 기본적인 진료를 위해 방문하겠다 |  |  |  |
| 간단한 시술(상처소독/피부봉합 등)을 위해 방문하겠다 |  |  |  |
| 식사/운동/흡연/음주 등 건강관리를 위해 상담 받겠다 |  |  |  |
| 정기적인 건강검진을 위해 방문하겠다 |  |  |  |
| 의사가 나의 중요한 병력과 건강문제를 잘 파악하고 있다 |  |  |  |
| 추가 검사나 치료 필요 시 전문의사를 적절히 추천해준다 |  |  |  |
| 의사가 나의 말과 질문을 경청하고 잘 이해한다 |  |  |  |
| 나의 건강상태와 검사결과를 쉽고 자세히 잘 설명해준다 |  |  |  |
| 나의 신체건강뿐만 아니라 정신건강에도 관심을 보인다 |  |  |  |
| 내 가족과 생활환경을 잘 파악하고 관심을 기울인다 |  |  |  |
| 지역사회 건강증진에도 기여한다(건강강좌/방문간호 등) |  |  |  |
| 서비스 개선을 위해 환자의견을 반영한다(만족도설문 등) |  |  |  |
| 진단과 치료에 대한 의사의 결정을 신뢰할 수 있다 |  |  |  |
| 제공되는 의료서비스에 대해 전반적으로 만족한다 |  |  |  |
