# Supplementary material for: Comparison of patient perceptions of primary care quality across healthcare facilities in Korea: A cross-sectional study
Source: PLoS One. 2020 Mar 10;15(3):e0230034. doi: 10.1371/journal.pone.0230034 (PMC7064208; doi:10.1371/journal.pone.0230034)
Supplement: S1 Table — K-PCAT = Korean Primary Care Assessment Tool, β = regression coefficient, SE = standard errors, UHS = university health service Statistically significant results are marked in bold. a Includes hypertension, diabetes, dyslipidemia, heart disease, hyperuricemia, chronic viral hepatitis, arthritis, cancer, depression, and anxiety disorder b Out-of-pocket payment only. KRW, Korean Won. 10,000 KRW ≈ 8.85 USD. (DOCX) [file pone.0230034.s004.docx]

**S1 Table. Factors Associated with K-PCAT Total Scores for Each Healthcare Facility**

|  | **UHS (n = 5,453)** | | | |  | **Clinic (n = 5,519)** | | | |  | **Hospital (n = 5,014)** | | | |
| --- | --- | --- | --- | --- | --- | --- | --- | --- | --- | --- | --- | --- | --- | --- |
|  | **β** | **SE** | ***P*** | ***P*_trend_** |  | **β** | **SE** | ***P*** | ***P*_trend_** |  | **β** | **SE** | ***P*** | ***P*_trend_** |
| Age | -0.07 | 0.03 | **.029** |  |  | -0.28 | 0.03 | **<.001** |  |  | -0.29 | 0.03 | **<.001** |  |
| Sex |  |  |  |  |  |  |  |  |  |  |  |  |  |  |
| Male | (ref) | – |  |  |  | (ref) | – |  |  |  | (ref) | – |  |  |
| Female | -3.77 | 0.43 | **<.001** |  |  | -1.45 | 0.35 | **<.001** |  |  | -3.56 | 0.41 | **<.001** |  |
| Job |  |  |  |  |  |  |  |  |  |  |  |  |  |  |
| Student | (ref) | – |  |  |  | (ref) | – |  |  |  | (ref) | – |  |  |
| Staff | -5.51 | 0.70 | **<.001** |  |  | 0.34 | 0.56 | .541 |  |  | -0.03 | 0.66 | .968 |  |
| Faculty | -3.74 | 0.97 | **<.001** |  |  | 0.44 | 0.77 | .568 |  |  | 1.57 | 0.89 | .079 |  |
| Income level |  |  |  | .834 |  |  |  |  | .262 |  |  |  |  | **<.001** |
| Low | (ref) | – |  |  |  | (ref) | – |  |  |  | (ref) | – |  |  |
| Middle | 0.33 | 0.59 | .576 |  |  | 0.17 | 0.48 | .720 |  |  | 2.01 | 0.57 | **.001** |  |
| High | -0.02 | 0.63 | .976 |  |  | 0.53 | 0.51 | .302 |  |  | 3.10 | 0.61 | **<.001** |  |
| Self-perceived health |  |  |  | **.004** |  |  |  |  | **<.001** |  |  |  |  | .328 |
| Good | (ref) | – |  |  |  | (ref) | – |  |  |  | (ref) | – |  |  |
| Fair | -1.46 | 0.47 | **.002** |  |  | -1.60 | 0.38 | **<.001** |  |  | -0.40 | 0.45 | .369 |  |
| Poor | -1.44 | 0.89 | .105 |  |  | -2.62 | 0.72 | **<.001** |  |  | -0.59 | 0.84 | .481 |  |
| Comorbidity |  |  |  |  |  |  |  |  |  |  |  |  |  |  |
| None | (ref) | – |  |  |  | (ref) | – |  |  |  | (ref) | – |  |  |
| Acute disease only | -0.49 | 0.57 | .389 |  |  | -0.11 | 0.46 | .806 |  |  | -0.16 | 0.55 | .771 |  |
| Chronic condition^a^ | 0.07 | 0.65 | .912 |  |  | -0.97 | 0.53 | .066 |  |  | -0.66 | 0.62 | .290 |  |
| Hospital visit for chronic condition |  |  |  |  |  |  |  |  |  |  |  |  |  |  |
| No | (ref) | – |  |  |  | (ref) | – |  |  |  | (ref) | – |  |  |
| Yes | 0.30 | 0.43 | .487 |  |  | 0.63 | 0.34 | .066 |  |  | -0.13 | 0.41 | .743 |  |
| Medical doctor in the family |  |  |  |  |  |  |  |  |  |  |  |  |  |  |
| No | (ref) | – |  |  |  | (ref) | – |  |  |  | (ref) | – |  |  |
| Yes | -1.77 | 0.74 | **.016** |  |  | 0.03 | 0.60 | .958 |  |  | 0.54 | 0.69 | .437 |  |
| Having a regular doctor |  |  |  |  |  |  |  |  |  |  |  |  |  |  |
| No | (ref) | – |  |  |  | (ref) | – |  |  |  | (ref) | – |  |  |
| Yes | 1.69 | 0.63 | **.007** |  |  | 4.28 | 0.51 | **<.001** |  |  | 4.06 | 0.59 | **<.001** |  |
| Ambulatory care visit per year, n |  |  |  | **.002** |  |  |  |  | .107 |  |  |  |  | **<.001** |
| 0–3 | (ref) | – |  |  |  | (ref) | – |  |  |  | (ref) | – |  |  |
| 4–6 | 0.55 | 0.53 | .303 |  |  | 0.32 | 0.43 | .464 |  |  | -1.03 | 0.51 | **.043** |  |
| 7–12 | 1.73 | 0.77 | **.024** |  |  | -0.12 | 0.62 | .851 |  |  | -2.13 | 0.73 | **.003** |  |
| ≥13 | 3.12 | 1.06 | **.003** |  |  | 2.15 | 0.85 | **.012** |  |  | -2.72 | 0.99 | **.006** |  |
| Medical expense per year, KRW^b^ |  |  |  | **.022** |  |  |  |  | .100 |  |  |  |  | **<.001** |
| <250,000 | (ref) | – |  |  |  | (ref) | – |  |  |  | (ref) | – |  |  |
| 250,000–499,999 | -0.83 | 0.67 | .213 |  |  | 0.39 | 0.54 | .471 |  |  | 2.55 | 0.63 | **<.001** |  |
| 500,000–999,999 | -1.06 | 0.83 | .205 |  |  | -0.82 | 0.67 | .223 |  |  | 2.20 | 0.77 | **.005** |  |
| ≥1,000,000 | -2.31 | 1.08 | **.032** |  |  | -1.47 | 0.87 | .091 |  |  | 2.16 | 1.00 | **.032** |  |

K-PCAT = Korean Primary Care Assessment Tool, β = regression coefficient, SE = standard errors, UHS = university health service

Statistically significant results are marked in bold.

^a^ Includes hypertension, diabetes, dyslipidemia, heart disease, hyperuricemia, chronic viral hepatitis, arthritis, cancer, depression, and anxiety disorder

^b^ Out-of-pocket payment only. KRW, Korean Won. 10,000 KRW ≈ 8.85 USD
