# Supplementary material for: Comparison of patient perceptions of primary care quality across healthcare facilities in Korea: A cross-sectional study
Source: PLoS One. 2020 Mar 10;15(3):e0230034. doi: 10.1371/journal.pone.0230034 (PMC7064208; doi:10.1371/journal.pone.0230034)
Supplement: Attachment — Submitted filename: Response to Reviewers.HKJOH.20200205.docx [file pone.0230034.s005.docx]

Dear Editor,

We deeply appreciate the opportunity to revise and re-submit our manuscript, titled **“Comparison of patient perceptions of primary care quality across healthcare facilities in Korea: a cross-sectional study”** to *PLOS ONE*. The reviewers' comments and suggestions were highly valuable in improving the quality of our manuscript. Below, we have addressed the editor’s and reviewers’ concerns in a point-by-point manner. We hope that the revised manuscript meets with your approval for publication.

Again, thank you very much for your thorough review of our manuscript.

Sincerely,

Hee-Kyung Joh

Seoul National University Health Service Center

1 Gwanak-ro, Gwanak-gu, Seoul, 08826, Republic of Korea

Telephone number: +82-2-880-5330

Fax number: +82-2-880-9274

E-mail address: hkjoh@snu.ac.kr

**Responses to academic editor:**

**1. Please ensure that your manuscript meets PLOS ONE's style requirements, including those for file naming. The PLOS ONE style templates can be found at http://www.plosone.org/attachments/PLOSOne_formatting_sample_main_body.pdf and http://www.plosone.org/attachments/PLOSOne_formatting_sample_title_authors_affiliations.pdf**

🡺 Per the editor’s comment, we have modified our manuscript and file names to meet the PLOS ONE’s style requirements.

**2. Please include additional information regarding the survey or questionnaire used in the study and ensure that you have provided sufficient details that others could replicate the analyses. For instance, if you developed a questionnaire as part of this study and it is not under a copyright more restrictive than CC-BY, please include a copy, in both the original language and English, as Supporting Information.**

🡺 Per the editor’s comment, we have included a copy of the questionnaire that we used in our study as Supporting Information (S1 and S2 Files) in both the original language and English.

**3. Please include captions for your Supporting Information files at the end of your manuscript, and update any in-text citations to match accordingly. Please see our Supporting Information guidelines for more information: http://journals.plos.org/plosone/s/supporting-information.**

🡺 Per the editor’s comment, we have included captions for the Supporting Information files at the end of our manuscript (page 27), and updated in-text citations to match accordingly (page 8, line 103; page 13, line 193).

**4. We note that you have indicated that data from this study are available upon request. PLOS only allows data to be available upon request if there are legal or ethical restrictions on sharing data publicly. For information on unacceptable data access restrictions, please see http://journals.plos.org/plosone/s/data-availability#loc-unacceptable-data-access-restrictions.**

**In your revised cover letter, please address the following prompts:**

**a) If there are ethical or legal restrictions on sharing a de-identified data set, please explain them in detail (e.g., data contain potentially identifying or sensitive patient information) and who has imposed them (e.g., an ethics committee). Please also provide contact information for a data access committee, ethics committee, or other institutional body to which data requests may be sent.**

**b) If there are no restrictions, please upload the minimal anonymized data set necessary to replicate your study findings as either Supporting Information files or to a stable, public repository and provide us with the relevant URLs, DOIs, or accession numbers. Please see http://www.bmj.com/content/340/bmj.c181.long for guidelines on how to de-identify and prepare clinical data for publication. For a list of acceptable repositories, please see http://journals.plos.org/plosone/s/data-availability#loc-recommended-repositories.**

**We will update your Data Availability statement on your behalf to reflect the information you provide.**

🡺 We acknowledge that there are no restrictions on sharing the de-identified data set that we have uploaded as a Supporting Information file (S1 Data). We have indicated this information in the revised cover letter.

**Responses to Reviewer #1**

**1. The manuscript is of great relevance to public health. But I believe the discussion could be more robust, given the data presented.**

🡺 We appreciate the reviewer’s comment. Per the reviewer’s suggestion, we have revised our Discussion section to make it more solid and based on our study results. We also have reorganized the paragraphs and have added more discussions on outpatient hospital care. The following paragraphs are one example: “Globally, the importance of providing healthcare at the right time and place, without compromising on care quality, is being emphasized.[3] Unnecessary utilization of hospital outpatient care is one of the main contributors of escalating healthcare costs.[25] Substituting hospital outpatient care with primary care is recommended as a solution to accomplish efficient and cost-effective healthcare delivery.[1,3] Evidence supports this perspective, indicating shorter waiting lists, shorter clinic waiting times, and higher patient satisfaction after the substitution interventions.[25]

Nonetheless, we found that the levels of trust and user satisfaction were highest for hospitals. This user view might be partly linked to the belief that good facilities and equipment of large hospitals guarantee good qualities of care.[13,23] Similar user misperception and distorted health-seeking behaviors were also reported in China; community individuals preferred to seek care in second or tertiary hospitals rather than primary care facilities, although the latter provide more accessible and affordable care. An important driver for this health-seeking behavior was trust in doctors in tertiary hospitals.[13] Therefore, efforts to raise public trust in primary care physicians and facilities are essential.” (Manuscript file, page 19–20, line 292–306)

Please refer to the ‘Manuscript’ file (page 16–21) and ‘Revised Manuscript with Track Changes’ file (page 16–22) to check the entire revision of the Discussion section. We have highlighted in yellow for the newly added parts in the Revised Manuscript with Track Changes file.

**2. In the discussion the main results are presented unnecessarily as they are all available in the "results" field.**

🡺 Per the reviewer’s comment, we have summarized the main results as concise as possible in the revised version. In addition, we have moved the following sentences to the Method section: “A university health service is a primary care model on campus dealing with medical care and health promotion of university students and employees. The university health service in this university implements a wide range of activities: primary medical consultation for acute and chronic diseases, mental health care, communicable disease prevention, immunizations, health screening, and health education. Family physicians play the central role in these activities, partly supported by some specialty care.”(Manuscript file, page 7, line 94–99)

**3. A large number of articles used in the discussion have more than 10 years of publication (33.0%). The inclusion of more current articles is necessary.**

🡺 Per the reviewer’s comment, we have additionally reviewed more recent studies and articles. In the revised version, 16 papers have been newly added, and 13 of them were published within 10 years. Overall, 28 of 40 (70%) references were published within 10 years, and 11 references within 2 years.

**4. I suggest discussing about the final sample size, 16.9% of the sample recruited. In addition to this issue, the sample was restricted to university users. Could these questions have influenced the results? Was the final sample size representative? The fact that they are all from university may have influenced the positive result for the service offered in this establishment? Can these questions be a limitation of the study?**

🡺 We agree with the reviewer’s point. Per the reviewer’s suggestion, we have added this point in the limitations as follows: “Fourth, we cannot rule out sampling bias. Because our sample was recruited from a university, this might have influenced the positive results for the university service. Although we did not restrict our sample to the users of the university health service to decrease such bias, our sample size (16.9% of the study population) and the non-random sampling method could have limited its representativeness.” (Manuscript file, page 21, line 323–327)

**5. I also suggest reviewing the formatting of the text and reviewing the writing of very long paragraphs.**

🡺 We appreciate the reviewer’s comment. Per the reviewer’s suggestion, the Discussion section was re-formatted. We have re-organized paragraphs to be shorter, using several subtitles, to enhance readers’ readability. Also, long sentences were trimmed as concisely as possible. Please refer to the ‘Manuscript’ file (page 16–21) and ‘Revised Manuscript with Track Changes’ file (page 16–22) to check the entire revision of the Discussion section.
